# 

**Published:** 1893-11

**Authors:** 


					﻿
ESTABLISHED 1855.	SUBSCRIPTION,
ATLANTA	Qt.
MEDICAL AND SURGICAL
JOURNAL.	I
se'v^’kS.'vJLAtlanta. Ga.. November. 1S93. No. 9M •
LUTHER B. GRANDY, M. 1).,	M. B. HUTCHINS, M. D.,
MANAGING EDITOR.	BUSINESS MANAGER.
				

